# Cold-induced dishabituation in rodents exposed to recurrent hypoglycaemia

**DOI:** 10.1007/s00125-021-05425-3

**Published:** 2021-03-17

**Authors:** Keeran Vickneson, Jessica Blackburn, Jennifer R. Gallagher, Mark L. Evans, Bastiaan E. de Galan, Ulrik Pedersen-Bjergaard, Bernard Thorens, Alison D. McNeilly, Rory J. McCrimmon

**Affiliations:** 1 School of Medicine, University of Dundee, Dundee, UK; 2 Division of Systems Medicine, School of Medicine, University of Dundee, Dundee, UK; 3 Wellcome Trust/MRC Institute of Metabolic Science, Cambridge, Cambridge, UK; 4 Radboud University Medical Center, Radboud University Nijmegen, Nijmegen, the Netherlands; 5 Maastricht University Medical Center+, Maastricht University, Maastricht, the Netherlands; 6 CARIM School for Cardiovascular Medicine, Maastricht University, Maastricht, the Netherlands; 7 Nordsjællands Hospital Hillerød, University of Copenhagen, Hillerød, Denmark; 8 Faculty of Biology and Medicine, University of Lausanne, Lausanne, Switzerland

**Keywords:** Cold, Counterregulatory responses, Habituation, Hypoglycaemia, Impaired awareness, Type 1 diabetes

## Abstract

**Aims/hypothesis:**

Recurrent hypoglycaemia in people with diabetes leads to progressive suppression of counterregulatory hormonal responses to subsequent hypoglycaemia. Recently it has been proposed that the mechanism underpinning this is a form of adaptive memory referred to as habituation. To test this hypothesis, we use two different durations of cold exposure to examine whether rodents exposed to recurrent hypoglycaemia exhibit two characteristic features of habituation, namely stimulus generalisation and dishabituation.

**Methods:**

In the first study (stimulus generalisation study), hyperinsulinaemic–hypoglycaemic (2.8 mmol/l) glucose clamps were performed in non-diabetic rodents exposed to prior moderate-duration cold (4°C for 3 h) or control conditions. In the second study (dishabituation study), rodents exposed to prior recurrent hypoglycaemia or saline (154 mmol/l NaCl) injections over 4 weeks underwent a longer-duration cold (4°C for 4.5 h) exposure followed 24 h later by a hyperinsulinaemic–hypoglycaemic (2.8 mmol/l) glucose clamp. Output measures were counterregulatory hormone responses during experimental hypoglycaemia.

**Results:**

Moderate-duration cold exposure blunted the adrenaline (epinephrine) response (15,266 ± 1920 vs 7981 ± 1258 pmol/l, Control vs Cold; *p* < 0.05) to next day hypoglycaemia in healthy non-diabetic rodents. In contrast, the suppressed adrenaline response (Control 5912 ± 1417 vs recurrent hypoglycaemia 1836 ± 736 pmol/l; *p* < 0.05) that is associated with recurrent hypoglycaemia was restored following longer-duration cold exposure (recurrent hypoglycaemia + Cold 4756 ± 826 pmol/l; not significant vs Control).

**Conclusions/interpretation:**

Non-diabetic rodents exhibit two cardinal features of habituation, namely stimulus generalisation and dishabituation. These findings provide further support for the hypothesis that suppressed counterregulatory responses following exposure to recurrent hypoglycaemia in diabetes result from habituation.

**Graphical abstract:**

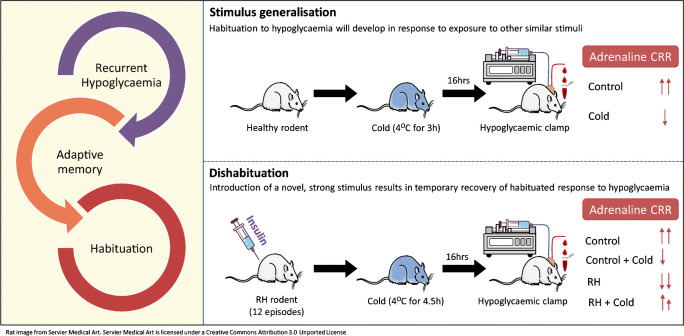

**Supplementary Information:**

The online version contains unedited but peer-reviewed supplementary material available at 10.1007/s00125-021-05425-3.

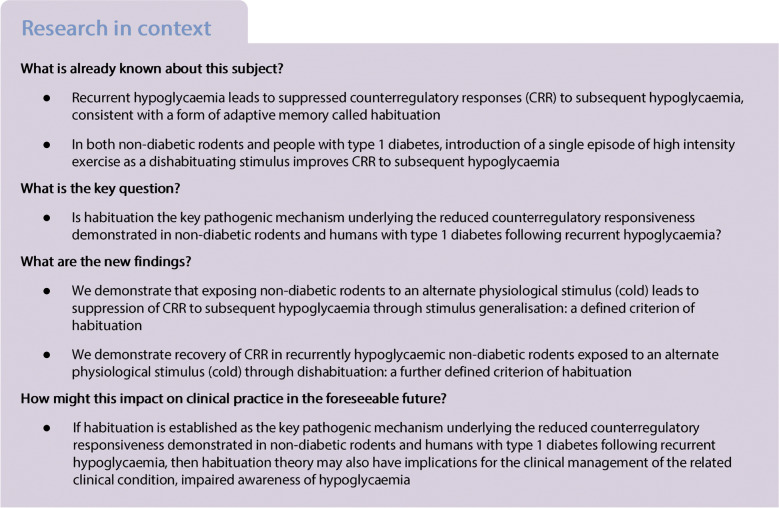



## Introduction

In type 1 diabetes, recurrent hypoglycaemia impairs symptomatic and hormonal counterregulatory responses (CRR) to subsequent hypoglycaemia [1, 2], culminating in a clinical condition referred to as impaired awareness of hypoglycaemia (IAH). IAH affects approximately 20–25% of all individuals with type 1 diabetes, increasing their risk of severe hypoglycaemia sixfold, and has a well-recognised morbidity burden [3]. The biological process through which recurrent hypoglycaemia leads to progressive suppression of hormonal CRR to subsequent hypoglycaemia remains unknown.

Habituation refers to a reduction in the psychological, behavioural and physiological responses to a stimulus as a result of repeated or prolonged exposure to that stimulus [4]. There are nine defined criteria of habituation, many of which are also seen in individuals with IAH (for review see [5]). Two of the these criteria are: (1) ‘stimulus generalisation’, where habituation to one stimulus will also develop in response to other stimuli that are similar to the original stimulus; and (2) ‘dishabituation’, where introducing an alternate and usually strong stimulus late in the habituation procedure can cause a temporary restoration of the habituated response [4]. Our group has recently shown in rodents exposed to recurrent hypoglycaemia [6], and humans with type 1 diabetes and IAH [7], that high intensity training (HIT) as a single alternate dishabituating stimulus successfully increased CRR during subsequent experimental hypoglycaemia. To further test the habituation hypothesis in the current study we examine whether an alternate physiological stimulus, cold exposure, can exhibit stimulus generalisation and dishabituation with hypoglycaemia.

## Methods

### Animals

Male Sprague Dawley rats (200–250 g, Charles River Laboratories, UK) were maintained on 12 h/12 h day/night cycle and provided with food and water ad libitum. Experimental procedures were approved by the University of Dundee Ethical Review Process and performed in accordance with UK Home Office regulations under the auspices of Project License PILPE82C1898.

### Experimental design

#### Experiment 1: Stimulus generalisation

**Fig. 1:**
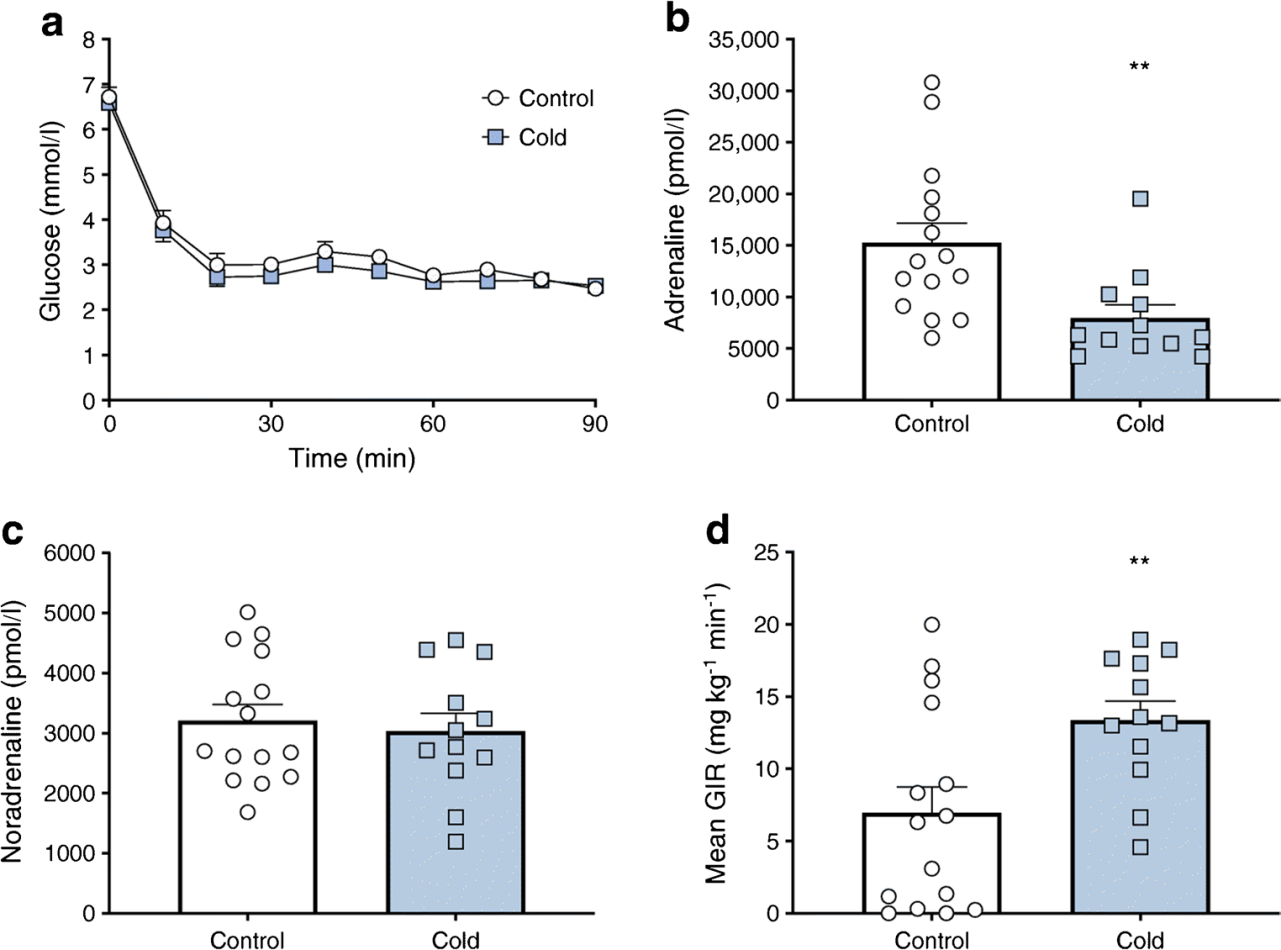
Stimulus generalisation with antecedent moderate cold exposure (4°C for 3 h) impairs CRR to subsequent hypoglycaemia. (**a**) Plasma glucose level during hyperinsulinaemic–hypoglycaemic clamp study on day 2. Plasma adrenaline (**b**) and noradrenaline (**c**) responses to stable hypoglycaemia. Mean exogenous GIR (**d**) during stable hypoglycaemia (60–90 min). Values shown are mean ± SEM. **p < 0.05.

Experimental procedures were performed over 2 consecutive days. On day 1, rodents were randomly assigned to either cold (4°C for 3 h) exposure (Cold; *n* = 12) or room temperature for 3 h in equivalent environment (Control; *n* = 15). On day 2, a hyperinsulinaemic–hypoglycaemic (2.8 mmol/l) clamp was performed with sampling of glucose hormones as previously described [8] (electronic supplementary material [ESM] Fig. 1a). The primary objective of Experiment 1 was to assess the effect of cold exposure on CRR to subsequent hypoglycaemia.

#### Experiment 2: Dishabituation

After 2 weeks of handling, animals were randomised to receive i.p. insulin-induced (0.5–1.0 U/kg i.p., NovoRapid; Novo Nordisk, Denmark) hypoglycaemia (recurrent hypoglycaemia; *n* = 19) or volume-matched saline (154 mmol/l NaCl) injections (Control; *n* = 19) 3 times weekly for 4 weeks (ESM Fig. 1b,c). Subsequently, vascular catheters were inserted under general anaesthesia as previously described [8]. Five days post-surgery, a further insulin or saline control injection was administered. On day 6, rodents were then randomised to receive either: (1) cold exposure (4°C for 4.5 h); or (2) no cold exposure in equivalent environment. The choice of 4.5 h cold-exposure duration was based on preliminary studies showing it provided a more robust physiological stimulus. On day 7, animals underwent a similar 90 min hyperinsulinaemic–hypoglycaemic (2.8 mmol/l) glucose clamp (ESM Fig. 1a). The primary objective of Experiment 2 was to assess the effects of antecedent cold exposure on subsequent CRR to hypoglycaemia in rodents habituated to hypoglycaemia.

In these studies, experimenters were not blinded to group assignment. Hormones were assayed by a technician blinded to group assignment. Only animals with non-functioning vascular catheters for hypoglycaemic clamp were excluded from the study.

### Counterregulatory hormone and metabolite analysis

Blood glucose levels were measured using Biosen (EFK Diagnostics, UK). Hormone levels were assessed as follows: insulin (multiplex ELISA; EMD Millipore Corporation, USA), adrenaline (epinephrine) (ELISA; Demeditec Diagnostics, Germany), noradrenaline (norepinephrine) (ELISA; Demeditec) and Brain-derived neurotrophic factor (BDNF ELISA; Biosensis, USA).

### Statistical analysis

Data were expressed as mean ± SEM. One-way ANOVA or repeated-measures ANOVA followed by Tukey post hoc correction for multiple comparisons to localise significant effects were used to assess between-group differences. *P* values less than 0.05 were considered statistically significant. All analyses were performed using Statistical Package for Social Sciences (SPSS) version 25 (IBM SPSS, USA) and GraphPad Prism version 7 (GraphPad Software, California, USA).

## Results

### Experiment 1: Stimulus generalisation

During day 2 hyperinsulinaemic–hypoglycaemic clamp, steady-state plasma glucose (2.6 ± 0.0 and 2.7 ± 0.1 mmol/l for Cold and Control groups respectively) were well matched between the two groups (Fig. 1a). In response to hypoglycaemia, the increase in plasma adrenaline (15,266 ± 1920 vs 7981 ± 1258 pmol/l, Control vs Cold; *p*< 0.05 [Fig. 1b]), but not plasma noradrenaline (Fig. 1c), was significantly attenuated by prior moderate-duration cold exposure. Consistent with suppression of the CRR, exogenous glucose infusion rate (GIR) was increased after antecedent cold exposure (7.0 ± 1.8 vs 13.4 ± 1.3 mg kg^−1^ min^−1^; *p* < 0.05) (Fig. 1d).

### Experiment 2: Dishabituation

**Fig. 2:**
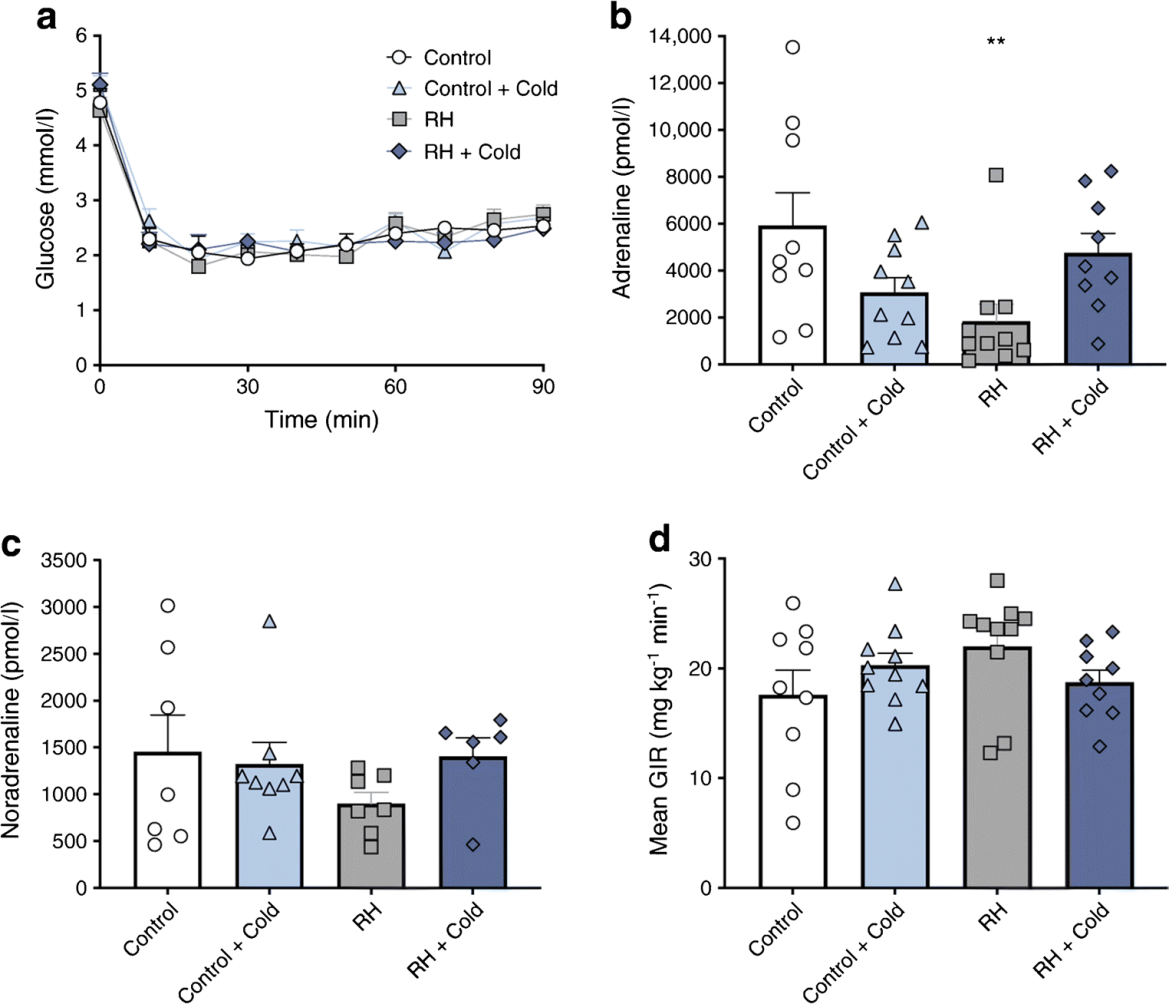
Dishabituation with acute strong cold exposure (4°C for 4.5 h) restores defective CRR to subsequent hypoglycaemia in a recurrent hypoglycaemia rodent model. (**a**) Plasma glucose level during hyperinsulinaemic–hypoglycaemic clamp study. Plasma adrenaline (**b**) and noradrenaline (**c**) responses during hypoglycaemia. (**d**) Mean exogenous GIR during stable hypoglycaemia (60–90 min). Values shown are mean ± SEM. **p < 0.05. RH, recurrent hypoglycaemia

Plasma glucose profiles were matched during the hypoglycaemic clamp and did not differ significantly (Fig. 2a). In Control animals, hypoglycaemia induced a pronounced adrenaline response, which, as expected, was markedly blunted following recurrent hypoglycaemia (Control 5912 ± 1417 vs recurrent hypoglycaemia 1836 ± 736 pmol/l; *p* < 0.05 [Fig. 2b]). In contrast, in recurrent hypoglycaemia rodents who had undergone the additional longer-duration cold exposure, adrenaline responses during the clamps study were restored (recurrent hypoglycaemia + Cold 4756 ± 826 pmol/l; difference not significant Control). Noradrenaline (Control 1451 ± 396 vs recurrent hypoglycaemia 900 ± 121 vs recurrent hypoglycaemia + Cold 1404 ± 197 pmol/l; [Fig. 2c]) and mean GIR (Control 17.6 ± 2.3 vs recurrent hypoglycaemia 22.0 ± 1.6 vs recurrent hypoglycaemia + Cold 18.7 ± 1.1 mg kg^−1^ min^−1^; [Fig. 2d]) showed similar patterns of change, but did not achieve statistical significance. Control (4 week saline-injected) animals exposed to cold displayed adrenaline responses that were reduced compared with non-exposed Control animals (3068 ± 632 pmol/l), consistent with stimulus generalisation despite the longer cold exposure.

## Discussion

Through demonstration of stimulus generalisation and dishabituation between two physiological stressors, cold and hypoglycaemia, this paper provides further evidence in support of the hypothesis that suppression of CRR following recurrent hypoglycaemia results from habituation. These findings, if supported by additional studies in people with type 1 diabetes and shown to also extend to psychological and behavioural responses to hypoglycaemia, may provide a framework for considering management strategies both for the avoidance and reversal of IAH.

In the present study, cold was chosen as an alternate physiological stimulus, as previous research had shown an alteration in stress responses to cold exposure in type 1 diabetes suggesting that there might be shared components within each homeostatic response [9]. Our findings support this hypothesis through demonstrating stimulus generalisation and dishabituation between these two physiological stressors. Moreover, because stimulus generalisation is thought to occur centrally, as opposed to a change in primary sensory afferents [10], our data suggest that habituation to recurrent hypoglycaemia may result from changes in key central nervous system integrative centres. However, the possibility that adaptations in peripheral organs (e.g. adrenal or peripheral sensors) also contribute to habituation cannot be excluded.

The different duration of cold exposure followed pilot studies showing the noradrenaline response to cold was augmented by 4.5 h vs 3 h exposure and it was anticipated a stronger stimulus would be required for dishabituation. However, in Experiments 1 and 2 both control groups exhibited stimulus generalisation, so this may not have been required and the difference in outcomes seems to have been determined more by prior habituation to hypoglycaemia than the strength of the alternate stimulus.

In contrast to stimulus generalisation, cold exposure increased CRR in rodents who had been habituated to hypoglycaemia. It is of note that the clear between-group differences in adrenaline response to subsequent hypoglycaemia were not reflected by significant differences in GIR (although the pattern of change was similar), which may be a result of the high insulin concentrations used in the clamp procedure attenuating those differences. Overall, the demonstration of a response to cold exposure in RH rodents that is consistent with cold dishabituation, combined with our previous study using HIT in rodents [6], lends further support to the hypothesis that the adaptation to recurrent hypoglycaemia is through the specialised form of adaptive memory, referred to as habituation.

This study has a number of limitations. First, while dishabituation leads to a restoration of the habituated response, it is possible that: (1) this effect is only transient; or (2) the individual may habituate to the dishabituating stimulus limiting the therapeutic utility of this approach [10]. An ongoing clinical study, HIT4HYPOS [11], will directly address this question through examining the impact of a 4 week HIT programme in people with type 1 diabetes who have impaired awareness of hypoglycaemia. Second, while we have studied two of the cardinal features of habituation, there remain a number of other criteria that need to be tested in order to establish habituation as the mechanism that underpins the development of IAH in humans. Third, a limitation of the rodent model is that it only enables assessment of CRR to hypoglycaemia, whereas people with IAH also demonstrate suppression of symptomatic, psychological and behavioural responses. Despite this, it should be recognised that mice, rats and other model systems all respond to recurrent hypoglycaemia in a very similar way to humans [5], and we have recently demonstrated in humans with long-standing type 1 diabetes the partial reversal of three important facets of IAH, namely hormonal, symptomatic and cognitive performance following a single episode of HIT [7].

In summary, in this paper we have demonstrated in the rodent model that dishabituation with cold exposure leads to, at least temporary, recovery of CRR to subsequent hypoglycaemia. Furthermore, we have demonstrated stimulus generalisability between cold and hypoglycaemia, providing further evidence that the reduced responsiveness to hypoglycaemia that follows recurrent exposure develops though habituation. This new understanding, if confirmed by other researchers, may lead to the development of novel approaches to the treatment of IAH.

### Supplementary Information

ESM (PDF 753 kb)

## Data Availability

Data are available on request from the corresponding author.

## Abbreviations

CRR: Counterregulatory responses
GIR: Glucose infusion rate
HIT: High intensity training
IAH: Impaired awareness of hypoglycaemia
