# Correction: Cold-induced dishabituation in rodents exposed to recurrent hypoglycaemia

**DOI:** 10.1007/s00125-024-06187-4

**Published:** 2024-06-03

**Authors:** Keeran Vickneson, Jessica Blackburn, Jennifer R. Gallagher, Mark L. Evans, Bastiaan E. de Galan, Ulrik Pedersen-Bjergaard, Bernard Thorens, Alison D. McNeilly, Rory J. McCrimmon

**Affiliations:** 1 School of Medicine, University of Dundee, Dundee, UK; 2 Division of Systems Medicine, School of Medicine, University of Dundee, Dundee, UK; 3 Wellcome Trust/MRC Institute of Metabolic Science, Cambridge, Cambridge, UK; 4 Radboud University Medical Center, Radboud University Nijmegen, Nijmegen, the Netherlands; 5 Maastricht University Medical Center+, Maastricht University, Maastricht, the Netherlands; 6 CARIM School for Cardiovascular Medicine, Maastricht University, Maastricht, the Netherlands; 7 Nordsjællands Hospital Hillerød, University of Copenhagen, Hillerød, Denmark; 8 Faculty of Biology and Medicine, University of Lausanne, Lausanne, Switzerland

**Correction: Diabetologia (2021) 64:1436-1441**

10.1007/s00125-021-05425-3

Unfortunately, the funding statement included in this paper did not fully satisfy the reporting requirements of the funder. The original article has been updated to include the sentence: ‘This paper reflects the authors’ views and the JU is not responsible for any use that may be made of the information it contains’.

