# Supplementary material for: Cold-induced dishabituation in rodents exposed to recurrent hypoglycaemia
Source: Diabetologia. 2021 Mar 17;64(6):1436–41. doi: 10.1007/s00125-021-05425-3 (PMC8099849; doi:10.1007/s00125-021-05425-3)
Supplement: Supplementary file 1 — (PDF 753 kb) [file 125_2021_5425_MOESM1_ESM.pdf]

Electronic Supplementary Figure 1:

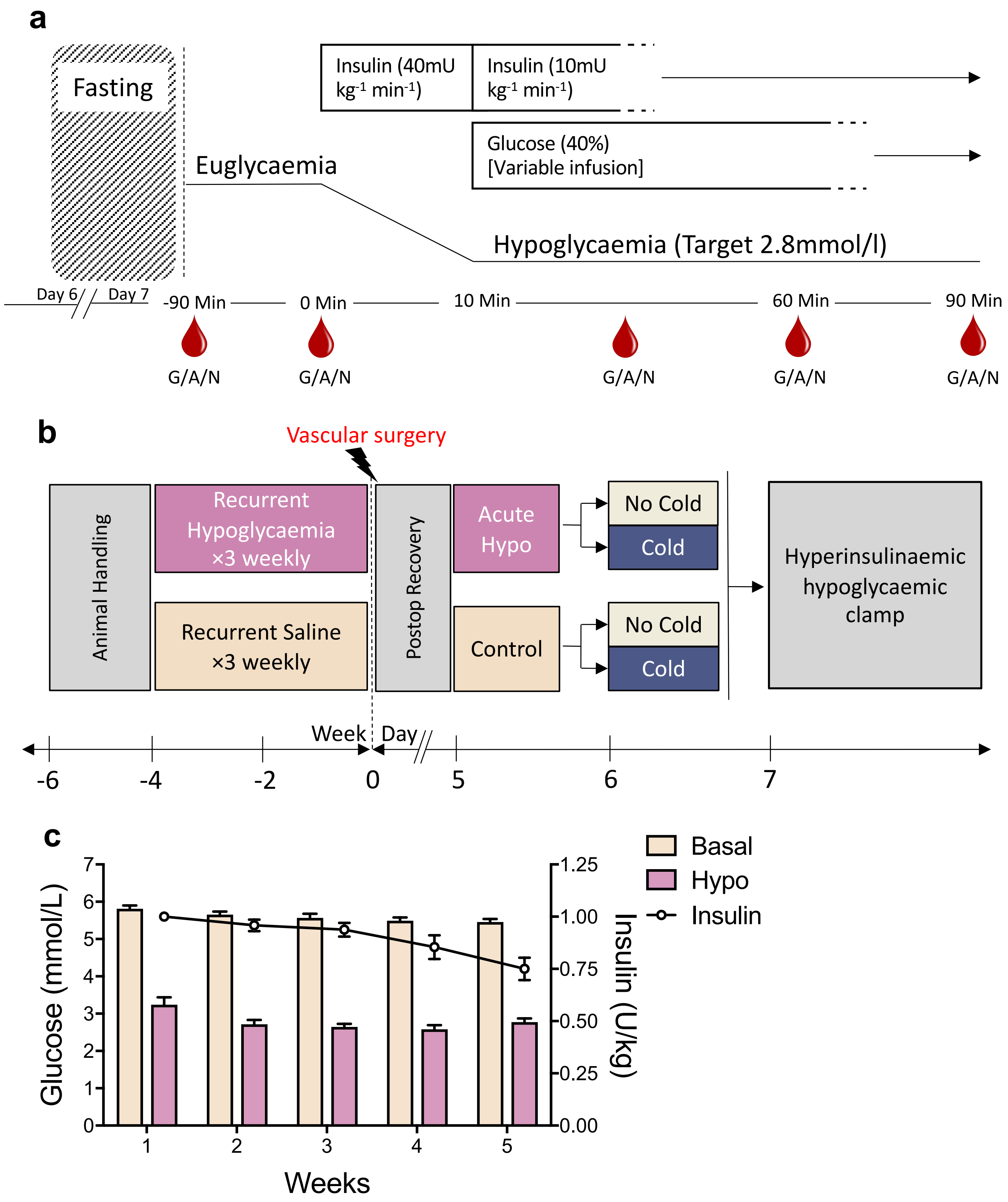

Study design. (a) Hyperinsulinaemic hypoglycaemic clamp protocol. Rodents were fasted overnight and vascular catheters were opened and flushed on the morning of the clamp. Animals were allowed to settle for at least 90 mins before a priming dose of insulin (40 mU kg^{-1} min^{-1}) was infused. After 10 mins, insulin infusion rate was stepped down to 10 mU kg^{-1} min^{-1} and a variable 40% dextrose infusion was adjusted based on bench-side glucose readings every 10 mins. Adrenaline (A) and noradrenaline (N) were sampled at baseline and at 30 min intervals during the clamp. **B:** Study design for Experiment 2. **C:** Insulin dose and blood glucose levels at baseline and during hypoglycaemia over 4 weeks of RH protocol.
